# Combination Model of Thyrotrophin Receptor Antibody and Volumetric Orbital Apex Crowding Index as an Indicator of Dysthyroid Optic Neuropathy

**DOI:** 10.1155/2021/9964232

**Published:** 2021-05-18

**Authors:** Zhihong Deng, Lu Chen, Jia Tan, Sha Wang, Dan Liu, Jinwei Wang, Chengzhi Jiang, Jie Yang, Bei Xu

**Affiliations:** ^1^Department of Ophthalmology, The Third Xiangya Hospital, Central South University, Changsha, Hunan, China; ^2^Eye Center of Xiangya Hospital, Central South University, Changsha, Hunan, China; ^3^Hunan Key Laboratory of Ophthalmology, Changsha, Hunan, China; ^4^PET-CT Center, Hunan Cancer Hospital, Changsha, Hunan, China

## Abstract

**Background:**

Dysthyroid optic neuropathy (DON) is one of the most serious vision-threatening complications of thyroid eye disease (TED); however, accurate and established diagnostic tools for DON are yet lacking. The present study was aimed at identifying new diagnostic factors for the accurate diagnosis of DON.

**Methods:**

This retrospective cross-sectional study included 25 TED patients (50 eyes) with enlarged extraocular muscles, no previous anti-inflammatory therapy, and the absence of other vision-affecting diseases between May 2017 and August 2019. Baseline data, such as gender, age, ophthalmological history, thyroid disease and management, TED history including clinical features, management, and long-term results, ophthalmological examinations, serology examinations, and single-photon emission computed tomography/computed tomography (SPECT/CT) results, were extracted. The diagnostic criteria were as follows: (1) best-corrected visual acuity (BCVA) loss coexisting with either of the following—increased latency or reduction of amplitude on visual evoked potential (VEP), impaired color vision, visual field defects, contrast sensitivity impairment, and optic disk swelling—and (2) Barrett′s index ≥ 60% in CT. Univariate and multivariate logistic regression analyses assessed the differences in age, gender, eyes, medical history, clinical activity, thyroid hormone and antibodies, uptake ratio (UR) of extraocular muscles in SPECT/CT, and volumetric orbital apex crowding index (VACI) using the generalized estimation equation. Consequently, the receiver operating characteristic curve (ROC) of the significant factors was constructed.

**Results:**

Univariate analysis revealed significant differences in the clinical activity, free triiodothyronine (FT3), free thyroxine (FT4), thyrotrophin receptor antibody (TRAb) levels, the UR of superior and medial rectus, and VACI between DON and TED (without DON) groups. Multivariate regression analysis revealed that TRAb and VACI were significantly different. ROC analysis showed that the univariate models of TRAb or VACI and the multivariate model were effective indicators of DON, while the multivariate model had the highest area under the ROC curve.

**Conclusion:**

A combination of TRAb and VACI is an effective indicator for DON.

## 1. Introduction

Thyroid eye disease (TED), also known as Graves' ophthalmopathy (GO) or thyroid-associated ophthalmopathy (TAO), is one of the most common autoimmune inflammatory disorders of the orbit [1]. The age-adjusted annual incidence of clinically relevant TED is 16 per 100,000 population in women and 2.9 in men [2]. TED is commonly associated with Graves' hyperthyroidism (Graves' disease) [3]. The pathological changes of TED affect periorbital connective tissue, extraocular muscles, and orbital fat tissue, causing ocular irritation, pain, double vision, and in some rare cases reduced vision and blindness [1].

Dysthyroid optic neuropathy (DON) is one of the rarest but most severe complications of TED, which occurs in approximately 3-8% of cases [4, 5]. The pathogenesis of the DON remains unclear. Previous studies have suggested that mechanical, vascular, and inflammatory components, secondary to an apex syndrome from hyaluronic acid production and adipogenesis enhanced by activated T cells and orbital fibroblasts, may lead to extraocular muscle enlargement and orbital fat expansion and overall increased vascular congestion [5, 6].

If not appropriately treated, DON may cause permanent vision loss; this has been observed in about 30% cases [7]. The current diagnosis of DON relies on the integrative analysis of multiple parameters by experienced clinicians as accurate and simple diagnostic tools are yet lacking. Previous studies have evaluated the clinical data and radiological findings associated with DON. Some of the risk factors associated with TED include age, gender, genetics, smoking, diabetes, thyroid dysfunction, and treatments for hyperthyroidism, which may exert a similar effect on DON [4, 5, 8–13]. However, the sensitivities and specificities of some visual function tests are not sufficient to predict DON. Barrett's index [14], the commonly used muscular index in the clinic to assess orbital apex crowding, cannot directly reflect the degree of muscle volume enlargement in the orbital apex. Therefore, the present study was aimed at identifying new sensitive indicators for detecting early optic nerve damage during the development of DON.

## 2. Materials and Methods

### 2.1. Patients

This was a single-center, retrospective study. All the TED patients at the Department of Ophthalmology, Xiangya Hospital, Central South University, Changsha, Hunan, China, between May 2017 and August 2019 were examined. This work has been carried out in accordance with the Declaration of Helsinki (2000) of the World Medical Association. This study was approved by the Ethic Committee of the Xiangya Hospital of Central South University, and all participants provided informed consent.

For each patient, we extracted the following data from the initial visit: gender and age, ophthalmological history, thyroid disease and managements, TED history, clinical features, management, and long-term results.

The ophthalmological examination included eye exophthalmos, best-corrected visual acuity (BCVA), slit-lamp examination, fundoscopy, visual evoked potential (VEP), color vision, visual field, and contrast sensitivity.

Inflammation was clinically evaluated using the clinical activity score (CAS) system by assessing two symptoms (ocular or retrobulbar pain and pain with eye movement) and five signs (conjunctival chemosis, conjunctival erythema, eyelid erythema, eyelid oedema, and swelling/erythema of the caruncle) for each eye. TED was confirmed “active” if CAS was ≥3 (0 to 7) [15].

Serology examination included free triiodothyronine (FT3), free thyroxine (FT4), thyroid-stimulating hormone (TSH), thyroglobulin antibody (TGA), thyroid peroxidase antibody (TPOA), and thyrotrophin receptor antibody (TRAb), which were detected using the electrochemiluminescence immunoassay (Roche Diagnostic Product Co., Ltd., Shanghai, China).

All patients underwent orbital single-photon emission computed tomography (SPECT)/CT (Philips, USA). Briefly, 555 MBq (15 mCi) ^99m^Tc- (Beijing Atomic High-Tech Co. Ltd., Beijing, China) DTPA (Jiangyuan Pharmaceutical Factory, Jiangsu Institute of Atomic Medicine, Jiangsu, China) was intravenously administered 20 min before imaging. A CT scan with 1 mm layer thickness was performed first, followed by SPECT imaging. Finally, the orbital CT image, SPECT image, and SPECT/CT fusion image were obtained using Philips image processing software.

### 2.2. Inclusion and Exclusion Criteria

The inclusion criteria were as follows: (1) patients who met the diagnostic criteria of TED [16] (eyelid retraction occurred in association with objective evidence of thyroid dysfunction or abnormal regulation, exophthalmos, optic nerve dysfunction, or extraocular muscle involvement. If eyelid retraction was absent, TED was diagnosed only if exophthalmos, optic nerve dysfunction, or extraocular muscle involvement was associated with thyroid dysfunction or abnormal regulation. The ophthalmic signs may be either unilateral or bilateral; confounding causes, including high myopia, congenital cranial-maxillofacial bone dysplasia, orbital tumor, and orbital nonspecific inflammation, were excluded) and (2) patients who reported to have enlarged extraocular muscles, as detected by SPECT/CT.

The exclusion criteria were as follows: (1) patients who received anti-inflammatory therapy before the first visit and (2) other causes that could explain the visual dysfunction, including cataracts, glaucoma, optic neuropathy, tear film abnormalities, and corneal changes.

Currently, there is no golden standard for the diagnosis of DON. In order to increase the reliability of the study, we referred to Saeed et al.'s clinical diagnostic flow [4] and set relatively stringent criteria. When DON was diagnosed simultaneously and independently by two senior ophthalmologists with at least 3-year experience in the treatment of the orbital disease, the eye was included in the DON group. The diagnostic criteria were as follows: (1) BCVA loss coexisting with either of the following—increased latency or reduction of amplitude on VEPs, impaired color vision, visual field defects, impaired contrast sensitivity, and optic disk swelling—and (2) Barrett′s index ≥ 60% in CT [14]. Then, the TED eye with normal vision was included in the TED group (without DON).

### 2.3. SPECT/CT Imaging Analysis

The uptake ratio (UR) of extraocular muscles and volumetric orbital apex crowding index (VACI) of each eye enrolled were calculated by a senior radiologist blinded to the grouping.

The regions of interest (ROI) of each extraocular muscle were delineated on the coronal plane, respectively. The mean value of the maximum count of the radioactivity in each extraocular muscle on the three levels was taken as the target value; the ROI in the occipital lobe was delineated on the cross section, and the average radioactive count was taken as the nontarget value. The UR was the target/nontarget ratio (T/NT).

The boundary of soft tissue in the orbital apex was recognized, automatically delineated, and manually modified using Philips image processing software according to a previously described approach [17]. The volume of the optic nerve was also manually removed to obtain the extraocular muscle volume. The ratio of the extraocular muscles' volume to the fat volume was calculated to determine the VACI.

### 2.4. Statistical Analysis

Statistical analysis was performed using IBM SPSS Statistics 24 (International Business Machines Corporation, USA). The normality of the distribution of continuous variables was tested by the Kolmogorov-Smirnov test. The continuous variables were described as mean values ± standard deviation (SD), while the categorical variables were described as numbers and percentages.

Both eyes of each patient were included in this study. The generalized estimation equation was used to increase the statistical power and account for the correlation between two eyes of an individual patient. All variables were assessed by univariate logistic regression analysis; those that were found to be associated (*P* < 0.1) were included in a multivariate model. Consequently, insignificant indicators were removed following a forward stepwise approach in the order of descending *P* values to reduce the collinearity issues.

The receiver operating characteristic curve (ROC) of the individual parameters within the final multivariate regression model and prognostic values of the multivariate model was developed. The areas under the ROC curves were calculated to descript the ability to indicate the presence of DON. The areas under the ROC curve from each model were then compared for statistical differences (*Z* test). The optimized Youden index determined the optimal cut points. The level of statistical significance was set at *P* < 0.05 for all the tests.

## 3. Results

A total of 50 eyes of 12 men (48%) and 13 women (52%) aged 31 to 69 years (mean 49.9 ± 9.9 y) with TED who met the inclusion criteria were included in the final analysis. Among those, 16 eyes of 11 patients were affected by DON, and 5 patients showed bilateral optic neuropathy. The contralateral eyes of 6 one-eye-affected patients were enrolled in the TED group (without DON), while 28 eyes of 14 TED patients without DON were enrolled in the TED group (without DON). The basic demographic data and clinical findings are summarized in Table 1.

The DON group consisted of more elders, males, and right eyes, compared with the TED group, but no statistical difference was observed with respect to age, sex, or lateral eyes.

The most common complaint of DON patients was vision loss and exophthalmos for TED (without DON) patients. The DON patients had a shorter history of thyroid dysfunction and eye complaints and a higher rate of I^131^ therapy than the TED group. On the other hand, most patients in both groups presented hyperthyroidism initially, but hypothyroidism was common in the DON group and hyperthyroidism in the TED group after an examination during the first visit at the eye clinic. However, the clinical history did not differ significantly between the two groups.

The BCVA of DON patients was logMAR (0.584 ± 0.591), which differed significantly from that of TED patients (−0.018 ± 0.055) (*P* < 0.05). The exophthalmos degree was 21.6 ± 4.1 mm for the DON group *vs*. 19.6 ± 2.7 mm for the TED group, with no statistical difference. The clinical activity score (CAS) was significantly different between the DON (4.17 ± 1.99) and TED groups (2.42 ± 1.14) (*P* = 0.026). Furthermore, 75% of DON patients and 38.2% without DON were in an active phase, but the difference was not significant.

Univariate regression analysis showed lower levels of free triiodothyronine (FT3), free thyroxine (FT4), and higher levels of TRAb in the DON group compared to the TED group (4.25 ± 1.25 pmol/L *vs*. 6.25 ± 2.49 pmol/L), 12.8 ± 4.4 pmol/L*vs*. 17.4 ± 4.9 pmol/L, and 27.0 ± 14.7 IU/L*vs*. 14.1 ± 11.8 IU/L, respectively; all *P* < 0.05). In addition, no significant difference was detected in TSH, TGA, or TPOA between the two groups (*P* > 0.05).

The UR of superior rectus was 10.2 ± 3.6 in the DON group *vs*. 8.0 ± 2.3 in the TED group, and the difference was significant (*P* < 0.05). However, no significant difference was detected in the UR of medial rectus, inferior rectus, lateral rectus, and mean ratio of the four recti between the two groups (*P* > 0.05).

Interestingly, VACI also showed a statistically significant difference between the groups in the univariate model (the means of DON and TED groups were 6.05 ± 3.16 and 2.42 ± 1.14, respectively; *P* < 0.05).

**Figure 1:**
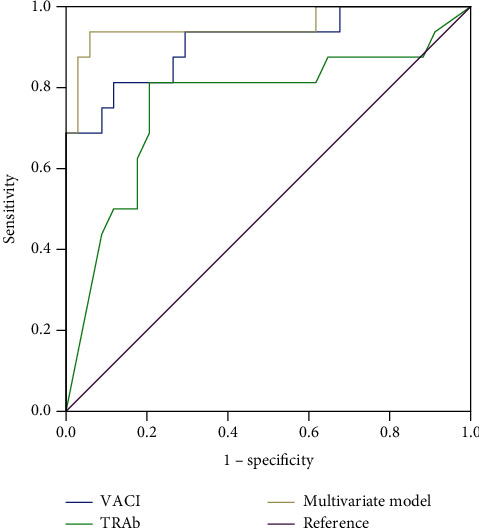
Receiver operating characteristic (ROC) curves of the multivariate model, volumetric orbital apex crowding index (VACI), and thyrotrophin receptor antibody (TRAb).

The significant factors of univariate analysis (*P* < 0.1), such as clinical activity, FT3, FT4, TRAb, UR of superior rectus and medial rectus, and VACI, were included in the multivariate model.  Table 2 lists the significant variables identified using multivariate regression with the generalized estimation equation. The two variables, VACI and TRAb, were associated with DON. Higher VACI and TRAb values suggested that the patient possibly was more likely to be suffering from DON. The equation was *Y* = −7.941.58 × VACI + 0.11 × TRAb. The area under the receiver operating characteristic (ROC) curve (AUC) for the predictive value (Pre = *e*^*Y*^/(1 + *e*^*Y*^)) of the multivariate model was 0.952, and that for the VACI and TRAb was 0.910 and 0.759, respectively (Figure 1 and Table 3). For the multivariate model, a cutoff of 0.31 (chosen according to an optimal Youden index of 0.879) yielded the best results with a sensitivity of 93.8% and a specificity of 94.1%; 94% of patients were correctly classified using this approach. Moreover, a cutoff of 3.11 in VACI (Youden index 0.695) resulted in a sensitivity of 81.3%, a specificity of 88.2%, and an accurate classification of 86% patients, while for the TRAb, a cutoff of 17.05 IU/L (Youden index 0.607) corresponded to a sensitivity and specificity of 81.3% and 79.4%, respectively, and an accurate classification of 80% of patients. The diagnostic accuracies of the multivariate model, VACI, and TRAb are listed in Table 4.

The AUCs from each model were compared to evaluate the statistical differences using the *Z* test (Table 5). The AUC of the multivariate model was significantly larger than that of VACI or TRAb, while the AUCs of VACI and TRAb did not differ significantly.

## 4. Discussion

For patients suffering from DON, which is the most serious vision-threatening complication related to TED, recognition is paramount for timely management [18]. In the present study, we assessed the factors such as age, gender, eyes, clinical history of thyroid disease and TED, type of thyroid disease, antithyroid therapy, degree of exophthalmos, clinical activity, thyroid hormone, antibody levels, UR of extraocular muscles in SPECT/CT, VACI, and the combination of parameters to find an efficient and reliable diagnostic test for DON.

The correlation between age and the development of DON has been reported previously [4] and proven by a multivariable model [8]; however, this association was not detected in our study. This contradiction was observed because some TED patients without extraocular muscle enlargement were excluded in this study.

Male gender was previously associated with DON by one univariate analysis [4], yet the correlation was not present in other preceding multivariable analysis [8] or the current study.

The differences between the two eyes of a patient had not been reported before and were also not analyzed statistically in this study. All the contralateral eyes of the 6 one-eye-affected patients have slight visual impairments (increased latency or reduction of amplitude on VEPs, impaired color vision, visual field defects, or contrast sensitivity impairment), but without BCVA loss, they were enrolled in the TED group (without DON). Owing to the successive onset of the bilateral eyes, quite a few of the TED patients present the condition in both eyes in different degrees. Therefore, the slightly visually impaired eyes might be set towards DON or already had potential optic neuropathy but were excluded based on normal BCVA. However, the diagnosis of the DON eye led to emergency treatments, such as high-dose intravenous glucocorticoids, which might stop the progress of the suspicious eye; however, whether these would convert into DON eventually cannot be judged.

Accumulating evidence indicated that I^131^ therapy is associated with an increased risk of occurrence or development of TED compared to antithyroid drugs (ATD) and thyroid surgery [19–21]. This phenomenon could result from the increased production of TRAb induced by radioactive iodine- (RAI-) associated leakage of thyroid antigens [19]. However, currently, there is no evidence, including our study, of its direct effect on DON.

Herein, we observed hyperthyroidism predominantly at the first visit in both groups, while the DON group had more hypothyroidism patients, without statistical difference. Both hyper- and hypothyroidism are associated with an elevated risk of progression or deterioration of TED, especially the hypothyroidism after I^131^ therapy for Graves' hyperthyroidism [21]. Nonetheless, the association between thyroid dysfunction and DON needs further investigation.

Thyroid antibodies are closely related to the development and severity of TED, especially the TSH receptor antibodies (TRAb). The expression of the TSH receptor is higher in TED orbital fat compared to that in normal orbital adipose tissues. TRA is a primary autoantigen involved in the pathophysiology of TED, increases the hyaluronic acid synthesis, and enhances adipogenesis in both orbital fibroblasts and preadipocytes [22]. Subsequently, the gradually increased orbital pressure leads to the compression and dysfunction of the optic nerve. Study results performed by several laboratories using different methodologies have identified the parallel between TRAb titers and the clinical features and the course of TED [23]. Ponto et al. [9] have identified TSI (thyroid-stimulating immunoglobulins, one of the four subtypes of TRAb) levels as a useful diagnostic tool for the identification of patients with early onset of DON. The current study confirmed this finding using total TRAbs with the Youden index of 0.607, suggesting that at the TRAb level > 17.05 IU/L, 81.3% TED patients might be at the risk of DON, while 21.6% TED patients without DON would be misdiagnosed.

The concentration influence of TSH, FT3, or FT4 on DON development has not yet been addressed by previous studies, nor have been the other thyroid antibodies, such as TPOA or TGA, which may also influence the extrathyroid manifestation. According to some studies, these disorders appear to predispose to worsening or development of TED; however, the contradictory results reported by other studies make this relationship difficult to interpret [21, 24]. In the present study, these factors did not result as indicators of DON in multivariable analysis.

The correlation of disease activity with the DON has been reported by Khong et al. [8]. Our univariate analysis also indicated that patients with a higher clinical activity score or uptake ratio of superior or medial Rectus were at higher risk of DON; however, when all explanatory variables including TRAb levels were considered, all of the activity indexes, including CAS, phases, or uptake ratios of extraocular muscles, were excluded as DON indicators. Several studies have shown a positive correlation between clinical activity and the levels of TRAb [25]; however, the TRAb levels were not included in Khong et al.'s study. In view of this, we assumed that TRAb levels dictated the incidence risk of DON, but not the clinical activities.

In TED patients, tissue expansion, especially the enlargement of extraocular muscles, occurs within the relatively fixed volume imposed by the bony orbit, resulting in orbital apex crowding, which has been demonstrated to be directly correlated with the development of DON. Nonetheless, to elucidate the degree of orbital apex crowding that leads to DON, accurate, quantizable, and repeatable tests are essential. Previous studies have explored several CT scan parameters for estimating the level of orbital apical crowding, such as linear or area measurements of extraocular muscles (on single coronal image), but volumetric estimates were proven to be a more useful indicator of DON [17]. In our study, 81.3% of TED patients with VACI > 3.11 were at the risk of DON, while 11.8% TED patients without DON were misdiagnosed as DON. Therefore, it could be speculated that other factors may be involved in the process (for example, optic nerve stretching). The cut point was lower than Goncalves' (4.14), which might be attributed to the exclusion of the optic nerve in the calculation of VACI.

The multivariate model of TRAb and VACI was a useful indicator of DON, with a larger AUC than the univariate models of TRAb or VACI. When the predictive value was >0.31, 93.8% of the patients were found to be suffering from DON, while only 5.9% without DON were misjudged.

## 5. Strengths and Limitations

Rather than age, gender, clinical history, thyroid disease, antithyroid therapy, clinical activity, exophthalmos, and other thyroid hormone and antibody levels, the high levels of VACI and TRAb should be the indicators of DON. These observations revealed that the combination of TRAb and VACI was a sensitive, objective, repeatable, and quantizable new indicator of DON, not contingent on expert experience. To the best of our knowledge, this is the first statistical confirmation of the association between this combined model and DON. Based on the predictive value calculated by TRAb and VACI, 94% of the patients could be correctly classified, and this accuracy was higher than that by TRAb or VACI alone.

The present study had several limitations. Firstly, the number of included patients was relatively small, especially in the DON group. Thus, a sufficient period is required to enlarge the sample size and increase the reliability of this study. Secondly, TSI, which is one of four subtypes of TRAb, showed a significant association with DON relative to other subtypes [25]. However, the detection of TSI has not yet been implemented in clinical practice in China and other countries, while TRAb is an accessible indicator.

In the future studies, we aim to assess a large sample size to increase the reliability, improve the automatic calculation program of the predictive value to report the risk of DON to doctors and patients directly, including the optic nerve stretching index to improve the model, and subsequently observe the dynamic changes in the treatment and improvement of DON.

## Figures and Tables

**Table 1:** Comparison of basic demographic data and clinical findings between TED patients with and without DON.

Parameter	DON group (16 eyes)	TED group (without DON) (34 eyes)	*P*
Demographic			
Male	8 (50.0%)	16 (47.1%)	0.870
Right eye	10 (62.5%)	15 (44.1%)	0.225
Age (years)	49.94 (11.48)	49.91 (9.29)	0.994
Clinical history			
Chief complaint			
Exophthalmos/vision loss/others	2/11/3	16/11/7	0.137
History of complaint (months)	7.3 (8.8)	9.4 (6.8)	0.480
Type of thyroid disease			
Hyperthyroidism/euthyroid	16/0	32/2	/
Antithyroid therapy			
ATD/I^131^	11/5	25/9	0.772
Thyroid function at the first visit			
Hyperthyroidism/euthyroid/hypothyroidism	3/4/9	17/9/8	0.103
History of thyroid dysfunction (months)	29.2 (28.6)	31.19 (49.4)	0.920
BCVA (logMAR)^∗^	0.584 (0.591)	-0.018 (0.055)	0.006
Degree of exophthalmos (mm)	21.6 (4.1)	19.6 (2.7)	0.213
Clinical activity			
CAS (0–7)^∗^	4.17 (1.99)	2.42 (1.14)	0.026
Active (CAS ≥ 3)	12 (75%)	13 (38.2%)	0.106
Thyroid hormone and antibodies			
FT3 (pmol/L)^∗^	4.25 (1.25)	6.25 (2.49)	0.033
FT4 (pmol/L)^∗^	12.8 (4.4)	17.4 (4.9)	0.029
TSH (mIU/L)	20.97 (33.43)	4.13 (7.75)	0.134
TGA (IU/mL)	1144 (1452)	578 (1237)	0.365
TPOA (IU/mL)	176 (173)	176 (191)	0.998
TRAb (IU/L)^∗^	27.0 (14.7)	14.1 (11.8)	0.009
Uptake ratio of extraocular muscles			
Medial rectus^∗∗^	12.7 (4.2)	10.4 (1.7)	0.070
Superior rectus^∗^	10.2 (3.6)	8.0 (2.3)	0.025
Inferior rectus	11.2 (4.0)	10.4 (2.2)	0.608
Lateral rectus	8.4 (3.6)	8.4 (2.0)	0.506
Mean of four recti	10.62 (3.64)	9.3 (1.3)	0.187
VACI^∗^	6.05 (3.16)	2.42 (1.14)	0.001

^∗^ *P* < 0.05, ^∗∗^*P* < 0.1. DON: dysthyroid optic neuropathy; TED: thyroid eye disease; ATD: antithyroid drug; CAS: clinical activity score (of TED); FT3: free triiodothyronine (reference range, 3.1–6.8); FT4: free thyroxine (reference range, 12–22); TSH: thyroid-stimulating hormone (reference range, 0.27–4.20); TGA: thyroglobulin antibody (reference range, 0–115); TPOA: thyroid peroxidase antibody (reference range, 0–34); TRAb: thyrotrophin receptor antibody (reference range, 0–1.75); VACI: volumetric orbital apex crowding index.

**Table 2:** Multivariate factors indicate the presence of dysthyroid optic neuropathy (DON).

Parameter	*B*	Std. error	95% profile likelihood confidence interval		Hypothesis test		
			Lower	Upper	Wald chi-square	df	Sig.
VACI	1.58	0.53	0.54	2.63	8.85	1	0.003
TRAb	0.11	0.05	0.02	0.20	5.87	1	0.015

TRAb: thyrotrophin receptor antibody; VACI: volumetric orbital apex crowding index.

**Table 3:** Parameters of receiver operating characteristic (ROC) curves of the multivariate model, volumetric orbital apex crowding index (VACI), and thyrotrophin receptor antibody (TRAb).

	The area under the ROC curve			Cutoff	Youden index	Sensitivity	Specificity
	Area	95% confidence interval					
VACI	0.910	0.815	1.000	3.11	0.695	81.3%	88.2%
TRAb	0.759	0.597	0.921	17.05	0.607	81.3%	79.4%
Multivariate model	0.952	0.876	1.000	0.31	0.879	93.8%	94.1%

**Table 4:** Diagnostic accuracies of the multivariate model, volumetric orbital apex crowding index (VACI), and thyrotrophin receptor antibody (TRAb).

		DON group	TED group (without DON)	Sum
TRAb	High	13 (true positive 81.3%)	7 (false positive 20.6%)	20
	Low	3 (false negative 18.8%)	27 (true negative 79.4%)	30
VACI	High	13 (true positive 81.3%)	4 (false positive 11.8%)	17
	Low	3 (false negative 18.8%)	30 (true negative 88.2%)	33
Multivariate model	(+)	15 (true positive 93.8%)	2 (false positive 5.9%)	17
	(-)	1 (false negative 6.3%)	31 (true negative 91.2%)	32
Sum		16	34	50

**Table 5:** *Z* test of receiver operating characteristic (ROC) curves of the multivariate model, volumetric orbital apex crowding index (VACI), and thyrotrophin receptor antibody (TRAb).

	*Z* value	*P*
VACI & multivariate model	2.44	0.007
TRAb & multivariate model	2.10	0.018
VACI & TRAb	1.57	0.058

## Data Availability

The data used to support the findings of this study are available from the corresponding author upon request.

## References

[1] Li Z., Cestari D. M., Fortin E. (2018). Thyroid eye disease: what is new to know?. *Current Opinion in Ophthalmology*.

[2] Hiromatsu Y., Eguchi H., Tani J., Kasaoka M., Teshima Y. (2014). Graves' ophthalmopathy: epidemiology and natural history. *Internal Medicine*.

[3] Weiler D. L. (2017). Thyroid eye disease: a review. *Clinical & Experimental Optometry*.

[4] Saeed P., Tavakoli Rad S., Bisschop P. H. L. T. (2018). Dysthyroid optic neuropathy. *Ophthalmic Plast Reconstr Surg*.

[5] Blandford A. D., Zhang D., Chundury R. V., Perry J. D. (2017). Dysthyroid optic neuropathy: update on pathogenesis, diagnosis, and management. *Expert Rev Ophthalmol.*.

[6] Bahn R. S. (2015). Current insights into the pathogenesis of Graves' ophthalmopathy. *Hormone and Metabolic Research*.

[7] Victores A. J., Takashima M. (2016 Winter). Thyroid eye disease: optic neuropathy and orbital decompression. *International Ophthalmology Clinics*.

[8] Khong J. J., Finch S., De Silva C. (2016). Risk factors for Graves' orbitopathy; the Australian Thyroid-Associated Orbitopathy Research (ATOR) Study. *The Journal of Clinical Endocrinology and Metabolism*.

[9] Ponto K. A., Diana T., Binder H. (2015). Thyroid-stimulating immunoglobulins indicate the onset of dysthyroid optic neuropathy. *Journal of Endocrinological Investigation*.

[10] Rutkowska-Hinc B., Maj E., Jabłońska A., Milczarek-Banach J., Bednarczuk T., Miśkiewicz P. (2018). Prevalence of radiological signs of dysthyroid optic neuropathy in magnetic resonance imaging in patients with active, moderate-to-severe, and very severe graves orbitopathy. *Eur Thyroid J.*.

[11] Lee H., Lee Y. H., Suh S. I., Jeong E. K., Baek S., Seo H. S. (2018). Characterizing intraorbital optic nerve changes on diffusion tensor imaging in thyroid eye disease before dysthyroid optic neuropathy. *Journal of Computer Assisted Tomography*.

[12] Wiersinga W. M. (2013). Smoking and thyroid. *Clinical Endocrinology*.

[13] Acharya S. H., Avenell A., Philip S., Burr J., Bevan J. S., Abraham P. (2008). Radioiodine therapy (RAI) for Graves' disease (GD) and the effect on ophthalmopathy: a systematic review. *Clinical Endocrinology*.

[14] Monteiro M. L. R., Gonçalves A. C. P., Silva C. T. M., Moura J. P., Ribeiro C. S., Gebrim E. M. M. S. (2008). Diagnostic ability of Barrett's index to detect dysthyroid optic neuropathy using multidetector computed tomography. *Clinics*.

[15] Barrio-Barrio J., Sabater A. L., Bonet-Farriol E., Velázquez-Villoria Á., Galofré J. C. (2015). Graves’ Ophthalmopathy: VISA versus EUGOGO Classification, Assessment, and Management. *Journal of Ophthalmology*.

[16] Bartley G. B., Gorman C. A. (1995). Diagnostic criteria for Graves' ophthalmopathy. *American Journal of Ophthalmology*.

[17] Gonçalves A. C., Silva L. N., Gebrim E. M., Matayoshi S., Monteiro M. L. (2012). Predicting dysthyroid optic neuropathy using computed tomography volumetric analyses of orbital structures. *Clinics (São Paulo, Brazil)*.

[18] Bartalena L., Baldeschi L., Boboridis K. (2016). The 2016 European Thyroid Association/European Group on Graves' orbitopathy guidelines for the management of Graves' orbitopathy. *European Thyroid Journal*.

[19] Ponto K. A., Zang S., Kahaly G. J. (2010). The tale of radioiodine and Graves' orbitopathy. *Thyroid*.

[20] Lantz M., Planck T., Asman P., Hallengren B. (2014). Increased TRAb and/or low anti-TPO titers at diagnosis of Graves' disease are associated with an increased risk of developing ophthalmopathy after onset. *Experimental and Clinical Endocrinology & Diabetes*.

[21] Stan M. N., Bahn R. S. (2010). Risk factors for development or deterioration of Graves' ophthalmopathy. *Thyroid*.

[22] Hesarghatta Shyamasunder A., Abraham P. (2017). Measuring TSH receptor antibody to influence treatment choices in Graves' disease. *Clinical Endocrinology*.

[23] Woo Y. J., Jang S. Y., Lim T. H. T., Yoon J. S. (2015). Clinical association of thyroid stimulating hormone receptor antibody levels with disease severity in the chronic inactive stage of Graves' orbitopathy. *Korean Journal of Ophthalmology*.

[24] Jarusaitiene D., Verkauskiene R., Jasinskas V., Jankauskiene J. (2016). Predictive Factors of Development of Graves’ Ophthalmopathy for Patients with Juvenile Graves’ Disease. *International Journal of Endocrinology*.

[25] Jang S. Y., Shin D. Y., Lee E. J., Yoon J. S. (2014). Clinical characteristics of Graves' orbitopathy in patients showing discrepancy between levels from TBII assays and TSI bioassay. *Clinical Endocrinology*.

